# Correction: Mamat @ Yusof et al. Expression of PD-1 and PD-L1 in Endometrial Cancer: Molecular and Clinical Significance. *Int. J. Mol. Sci.* 2023, *24*, 15233

**DOI:** 10.3390/ijms27209316

**Published:** 2026-10-20

**Authors:** Mohd Nazzary Mamat @ Yusof, Kah Teik Chew, Nirmala Chandralega Kampan, Mohamad Nasir Shafiee

**Affiliations:** Gynaecologic-Oncology Unit, Department of Obstetrics and Gynaecology, Hospital Canselor Tuanku Muhriz, Faculty of Medicine, Universiti Kebangsaan Malaysia, Kuala Lumpur 56000, Malaysia; p112039@siswa.ukm.edu.my (M.N.M.@Y.); nirmala@ppukm.ukm.edu.my (N.C.K.)

The journal’s Editorial Office and Editorial Board are jointly issuing a resolution and removal of the Journal Notice linked to this article [1]. Following concerns raised about the integrity of the peer-review (misconduct by one of two reviewers), the Editorial Office has conducted a post-publication peer-review of this article. This process included the recruitment of a new independent reviewer and was supervised by the original Academic Editor to ensure full compliance with MDPI’s Editorial Process (https://www.mdpi.com/editorial_process). The authors were unaware of misconduct by the reviewer, and had not included any suggestions by the reviewer for inappropriate references.

As a result of this process, the Academic Editor and the authors have agreed to update the following aspects of this publication:

Reviewer report 2 has been removed from the peer-review record, while one new review report has been added and uploaded to the peer-review record (https://www.mdpi.com/1422-0067/24/20/15233/review_report).

Based on the new review report, the following corrections have been made to the original publication:

1. The scope and limitations of the literature search have been clarified in Section 2. The following limitation has also been added:

“The literature search may have missed relevant studies because it did not systematically search for grey literature and non-English publications.”

2. Two sentences and their citations have been combined and revised as follows:

“The expression levels of PD-1 and PD-L1 in EC are remarkably high, with PD-1 being expressed in approximately 60–65% of EC cases and PD-L1 in a range of 25–70%, which are among the highest reported among gynaecological malignancies [19,83–88]. However, …”

3. Tumour cells and immune cells were spelled out throughout the text.

4. Table 2 has been added to the manuscript.

5. An Abbreviations section has been added.

**Abbreviations**
  The following abbreviations are used in this manuscript.
APCAntigen-presenting cellASCOAmerican Society of Clinical OncologyCPSCombined positive scoreCTLA-4Cytotoxic T-lymphocyte-associated protein 4DCsDendritic cellsdMMRDeficient mismatch repairECEndometrial cancerEMTEpithelial–mesenchymal transitionENGOTEuropean Network of Gynaecological Trial groupsESGOEuropean Society of Gynaecological OncologyESMOEuropean Society for Medical OncologyESPEuropean Society of PathologyESTROEuropean Society for Radiotherapy and OncologyFDAFood and Drug AdministrationFIGOInternational Federation of Gynecology and ObstetricsGLOBOCANGlobal Cancer ObservatoryICIsImmune checkpoint inhibitorsIDOIndoleamine 2,3-dioxygenaseIFN-γInterferon-gammaIHCImmunohistochemistryIRF9Interferon regulatory factor 9LVSILymphovascular space invasionMbMegabaseMMRMismatch repairMSIMicrosatellite instabilityMSI-HMicrosatellite instability-highMSSMicrosatellite stableNFATNuclear factor of activated T cellsNKNatural killerNSMPNo specific molecular profileORRObjective response rateOSOverall survivalPD-1Programmed cell death protein 1PD-L1Programmed death-ligand 1PD-L2Programmed death-ligand 2PFSProgression-free survivalPOLEDNA polymerase epsilonProMisEProactive Molecular Risk Classifier for Endometrial CancerSGOSociety of Gynecologic OncologySHP2Src homology region 2 domain-containing phosphatase 2STATSignal transducer and activator of transcriptionTCGAThe Cancer Genome AtlasTCRT-cell receptorTGF-βTransforming growth factor-betaVEGFVascular endothelial growth factor

With this update, the Academic Editor is satisfied that the editorial process related to this article has been completed as per MDPI’s Editorial Process policy. The Editorial Office would like to thank the authors for their collaboration during this process.

## Figures and Tables

**Table 2:** Summary of current clinical trials investigating immunotherapy-based strategies for endometrial cancer.

Therapy Regimen	Indication/Population	Efficacy	Reference
Nivolumab (anti-PD-1). Monotherapy	phase II, JapicCTI-163212. Advanced/recurrent corpus cancer	ORR 23%; median PFS 3.4 months; 6-month OS 73%; 12-month OS 49%.	Hasegawa et al., 2018 [111]
Avelumab (anti-PD-L1). Monotherapy	Phase II, Recurrent/persistent EC; evaluated according to MMR status	dMMR: ORR 26.7%; PFS 6-month 40%. Proficient MMR (pMMR): low activity; only 1/16 achieved both ORR and survival 6-month	Konstantinopoulos et al., 2019 [112]
Durvalumab (anti-PD-L1). Monotherapy	Phase II, PHAEDRA trial (ANZGOG1601). Advanced EC after 0–3 lines of chemotherapy, according to MMR status.	dMMR: ORR 47%, median PFS 5.5 months, 12-month OS 71%. pMMR: ORR 3%, PFS 1.8 months, 12-month OS 51%	Antill et al., 2019 [113]
Dostarlimab (anti-PD-1). Monotherapy	Nonrandomized Phase I, Recurrent/advanced dMMR EC after progression following platinum-based chemotherapy	ORR 42.3%; median PFS 8.1 months; DCR 57.7%	Oaknin et al., 2020 [114]
Lenvatinib + pembrolizumab (anti-PD-1). Combine therapy	Phase Ib/II. Previously treated advanced EC, including MSS/pMMR disease	Overall ORR 38.9%; overall median PFS 7.4 months; median OS 16.7 months; ORR PD-L1-positive tumour 35.8%; ORR PD-L1-negative tumour 39.5%; ORR MSS/pMMR 37.2%;ORR MSI-H/dMMR 63.6%; regardless MSI status, the median DOR—21.2 months, median PFS—7.4 months, median OS—16.7 months	Makker et al., 2020 [117]
Lenvatinib + pembrolizumab (anti-PD-1). Combine therapy	Phase III Study 309/KEYNOTE-775. Previously treated advanced/recurrent/metastatic EC, particularly pMMR disease, versus physician’s-choice chemotherapy	pMMR vs. chemotherapy: ORR 32.4% vs. 15.1%; OS 18.0 vs. 12.2 months; PFS 6.7 vs. 3.8 months All-comers vs. chemotherapy: ORR 33.8% vs. 14.7% %; OS 18.7 vs. 11.9 months; PFS 7.3 vs. 3.8 months	Makker et al., 2023 [118]
Cabozantinib + nivolumab (anti-PD-1) versus nivolumab. Combine therapy vs. monotherapy	Phase II, recurrent endometrial cancer.	Combination ORR 25%, median PFS 5.3 months vs. nivolumab alone ORR 16.7%, PFS 1.9 months	Lheureux et al., 2020 [119]
Pembrolizumab (anti-PD-1) or placebo along with combination therapy with paclitaxel plus carboplatin. Combine therapy.	Phase III, advanced or recurrent EC. Evaluated separately in dMMR and pMMR cohorts	dMMR: 12-month PFS 74% vs. 38% with chemotherapy alone; HR 0.30. pMMR: median PFS 13.1 vs. 8.7 months; HR 0.54.	Eskander et al., 2023 [120]
Dostarlimab (anti-PD-1) or placebo + carboplatin and paclitaxel. Combine therapy	Phase III, Primary advanced or recurrent EC	dMMR/MSI-H: 24-month PFS 61.4% vs. 15.7%, HR 0.28. (dostarlimab vs. placebo) Overall population: 24-month PFS 36.1% vs. 18.1%, HR 0.64; 24-month OS 71.3% vs. 56.0%. (dostarlimab vs. placebo)	Mirza et al., 2023 [121]
